# Low expression of PIK3C2A gene: A potential biomarker to predict the risk of acute myocardial infarction

**DOI:** 10.1097/MD.0000000000015061

**Published:** 2019-04-05

**Authors:** Buchuan Tan, Miao Liu, Yushuang Yang, Long Liu, Fanbo Meng

**Affiliations:** a China-Japan Union Hospital of Jilin University; b Cardiology Department of the China-Japan Union Hospital of Jilin University, Changchun, China.

**Keywords:** AMI, genetic marker, independent risk, peripheral blood, PIK3C2A

## Abstract

**Aims:**

Phosphoinositide 3-kinases (PI3Ks) are a family of enzymes that phosphorylate the 3′-OH of inositol ring of phosphatidylinositol (PI) and regulate a broad range of signaling pathways. PIK3C2A is structurally distinct from the other members of this class and is expressed in endothelial cells, vascular endothelium, and smooth muscle. In ischemic cardiovascular diseases, such as coronary artery disease, pathology is associated with endothelial damage and inflammation, downregulation of the EPC cell population and function, and impaired angiogenesis. This study aims to make an assessment on whether expression of PIK3C2A gene can be used as a biomarker for predicting the risk of acute myocardial infarction (AMI).

**Methods:**

We collected peripheral blood from 84 subjects with non-coronary heart disease and 70 patients with AMI. The real-time quantitative PCR test was applied to measure levels of PIK3C2A gene expression at mRNA level in peripheral blood.

**Results:**

Our results indicated that the level of PIK3C2A gene expression in peripheral blood of AMI patients was significantly lower than one in the non-coronary heart disease subjects. Binary logistic regression analysis showed that low expression of PIK3C2A gene was an independent risk factor of AMI and increased the risk of AMI by 2.231 folds. Moreover, it was found that low expression of PIK3C2A gene was not associated with level of fasting blood glucose, platelet count, Gensini score of coronary artery, and quantity of cardiac troponin.

**Conclusion:**

The level of PIK3C2A gene expression in patients with AMI is significantly lower than that of healthy people. Low expression of PIK3C2A gene is an independent risk factor of AMI. Low expression of PIK3C2A could serve as a potential biomarker to predict risk of AMI.

## 1 Introduction

Acute myocardial infarction (AMI) was the most severe coronary artery disease which caused more than 24 million deaths in the USA and more than 4 million deaths in Europe and northern Asia. More than one-third of these deaths occurred in developed countries annually.^[1,2]^ Increased applications of evidence-based therapies and lifestyle changes have dramatically reduced mortality rate of coronary heart disease in recent decades.^[1]^ In USA, the age- and sex-adjusted incidence of AMI has decreased over the last decade from 287 to 208 cases per 100,000 person-years. The consequently reduced mortality rate might be related to the improved primary and secondary prevention efforts. However, the mortality rate did not change in low-income countries.^[2,3]^ Accumulated evidence showed that smoking, diabetes, hyperlipidemia, and hypertension were all independent risk factors for CHD. The majority (85%–90%) of patients with premature CHD had at least one conventional risk factor.^[4,5]^ Epidemiological studies demonstrated that the treatments by controlling these risk factors might reduce the morbidity rate.^[5]^ As same as other chronic diseases, coronary heart disease (CHD) is a complex genetic disorder caused by the effects of multiple genes in combination with inherent and environmental factors and its incidence increases with age.^[6]^ Despite many risk factors for AMI, research targeting sensitive genes might provide better predictive and preventive strategies.

It was shown that levels of gene expression in peripheral blood might reflect changes of a number of complex diseases, including CHD, and could serve as an important genetic biomarker for detection and verification of diseases.^[7,8]^ The module composed of 22 genes, including ABCG2, ADIPOQ, ALPL, and CAV3, which might play a potential role in protecting arterioles and could predict the risk of hypertension among healthy people.^[9]^ The level of AdipoR2 mRNA expression in peripheral blood was related to the progression of coronary atherosclerosis.^[10]^ Over-expression of KIAA0101 mRNA in peripheral blood could be used as a predictor for invasion and progression of liver cancer.^[11]^ The p66^shc^gene expression in peripheral blood monocytes might be a strong indication of vascular injury in high-risk patients such as those with diabetes mellitus and/or CAD.^[12]^

Phosphoinositide 3-kinases (PI3Ks) were a family of enzymes that phosphorylated the 3′-OH position of the inositol ring of phosphatidylinositol (PI), and regulated signaling pathways.^[13]^ For example, the class I PI3K, p110β impaired platelet activation and prevented the formation of arterial thrombosis.^[14,15]^ In contrast to the class I PI3Ks, the roles of the class II PI3Ks (PI3KC2α, -C2β, and -C2γ) remained unknown. Recent studies showed that the lack of PIK3C2A altered the membrane structure in platelets and macrophages and thus played a regulatory role in platelet function sufficient to affect in vivo arterial thrombosis.^[13]^ PIK3C2A could also affect angiogenesis contributing to the pathophysiology of coronary artery disease.^[16]^ Furthermore, PIK3C2A gene could affect cell proliferation and migration.^[17]^ The lack of PIK3C2A gene expression also found to contribute to the progression of diabetes by affecting the proliferation and differentiation of pancreatic β cells.^[18]^ Our previous pilot study on profiling gene expression in peripheral blood of AMI patients indicated that AMI patients had a lower expression of PIK3C2A than non-CHD patients. In this study, we scale up our sample size to increase statistical power in order to further assess and validate the association between PIK3C2A gene expression and AMI.

## 2 Subjects and methods

### 2.1 Subjects

All the studied subjects were recruited from the patients admitted into Department of Cardiovascular Medicine, China-Japan Union Hospital of Jilin University from April 2016 to September 2016 and subjected to coronary angiography. Seventy subjects diagnosed as AMI according to the global definition of myocardial infarction issued in 2012^[19]^ and eighty-four subjects without CHD were selected as the AMI and the control groups, respectively. AMI patients met the inclusion criteria: MI Type 1: spontaneous myocardial infarction related to atherosclerotic plaque rupture, ulceration, assuring, erosion, or dissection with resulting intraluminal thrombus in one or more of the coronary arteries leading to decreased myocardial blood flow or distal platelet emboli with ensuing myocyte necrosis. The exclusion criteria used in this research were as follows:

(1) MI Type 2: myocardial infarction secondary to an ischemic imbalance;
(2) MI Type 3: myocardial infarction resulting in death when biomarker values are unavailable
(3) MI Type 4: myocardial infarction related to percutaneous coronary intervention or stent thrombosis;
(4) MI Type 5: myocardial infarction related to coronary artery bypass grafting (CABG).

### 2.2 Peripheral blood collection, total RNA extraction, and cDNA synthesis

Four milliliters of peripheral venous blood was collected from each subject followed by total RNA extraction using total RNA extraction reagent kit (RNAsimple Total RNA Kit, Tiangen Biotech Ltd., Beijing) according to the company's instruction. One microgram of the qualified total RNA was then subjected to reverse transcription using reverse transcription reagent kit (TOYOBO Rever Tra Ace qPRC RT kit, Shanghai) and the obtained cDNA sample was stored at −20°C for real-time quantitative PCR.

### 2.3 Real-time quantitative PCR

PCR amplification was carried out with SYBR real-time quantitative PCR reagent kit (SYBR Premix Ex Taq TM, TaKaRa, Dalian). In brief, 20 μl reaction system was adopted, in which each reaction included: 10 μl of SYBR Premix Ex Taq TM, 0.5 μl of forward primer and 0.5 μl of reverse primer (concentration: 10 μmol/L), 8 μl of nuclease-free double distilled water, and 1 μl of cDNA template. Amplification was realized by using Mx3005P real-time quantitative PCR system (Strata Gene). Relative expression quantity 2^−ΔCt^ (ΔCt = Target Gene Ct Value − Reference Gene Ct Value) was used to represent the obtained cycle thresholds (Ct) of each sample and a comparison was conducted.^[20]^ Design of PCR primer was conducted according to PIK3C2A gene sequence obtained from NCBI Genbank. Primer synthesis was completed by Shanghai-based Sangon Biotech.

### 2.4 Statistical analysis

All the statistical analysis was performed in SPSS 24.0. Both independent *t* test and rank-sum test were applied in comparing inter-groups. *χ*^2^ test was applied in determining the differences among groups. Binary logistic regression analysis was carried out to analyze relevant risk factors of AMI. As for the correlation of PIK3C2A with cardiac troponin I and Gensini, double-variable correlation analysis was applied. *P* value less than .05 was considered significant.

## 3 Results

### 3.1 Clinical characterization of the studied subjects

There was no significant difference between the two groups in terms of age, sex, BMI, history of hypertension, family history of coronary heart disease, systolic pressure, TG, TC, LDL-C, and HDL-C. Patients in the AMI group was obviously older than ones in the control group (*t* = −3.133, *P* = .002). There was a significant difference between the two groups in the fasting blood glucose (*Z* = −2.899, *P* = .004). There was also a significant difference between WBC counts in the two groups (*t* = 3.933, *P* = .000). There was no significant difference of dose of the taken asprin, clopidogrel, and ticagrelor between the two groups. There was a significant difference of dose of anticoagulants taken between the two groups (see Table 1).

**Table 1:**
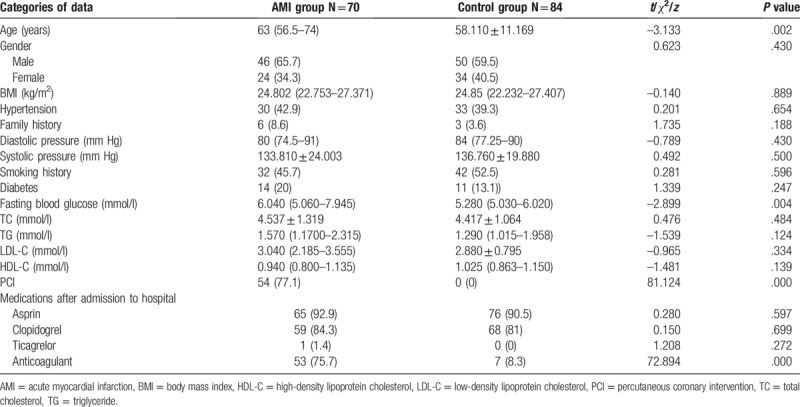
Comparison of clinical data between the AMI and the control groups.

### 3.2 Identification of amplified product by real-time fluorescence quantitative PCR for PIK3C2A gene

The result of real-time fluorescence quantitative PCR test on RNA of peripheral blood indicated that the amplification curve of PIK3C2A gene had an obviously smooth “S-shape”. Dissociation curve had a single dissociation peak and amplified product had a relatively high degree of specificity.

### 3.3 PIK3C2A mRNA expression in the AMI and non-CHD groups

**Figure 1:**
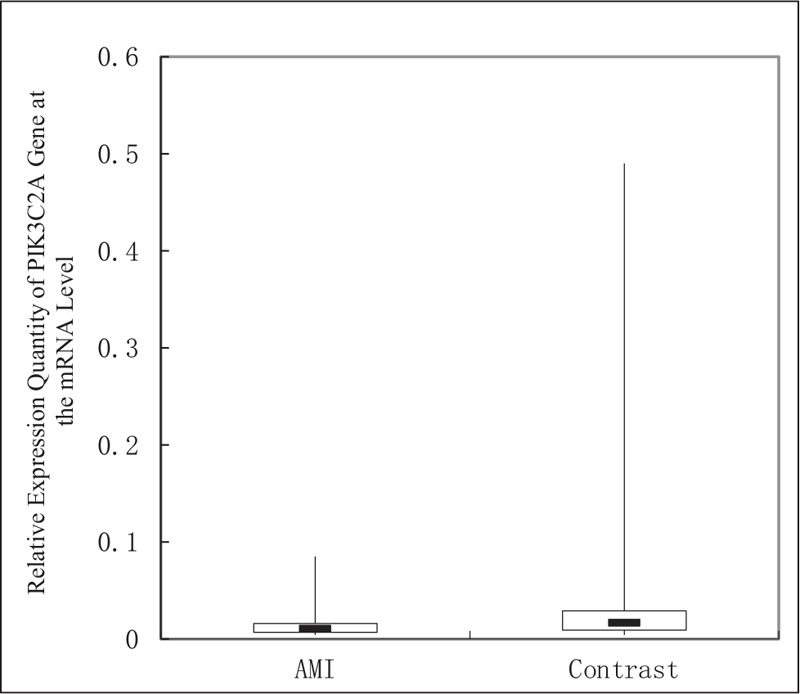
Comparison of relative expression quantity of PIK3C2A gene at the mRNA level between the AMI group and the control groups. AMI = acute myocardial infarction.

It was shown that 2^−ΔCT^ of the AMI group is 0.011 (0.007–0.016) and 2^−ΔCT^ of the control group is 0.013 (0.009–0.029), and the difference was significant between the two groups (*Z* = −2.606, *P* = .012). In peripheral blood of the AMI group, the level of PIK3C2A mRNA was significantly lower than that of the control group. The relative expression quantity of PIK3C2A gene in the AMI group was 0.85 times of that in the control group. See Figure 1 for details.

### 3.4 Association between PIK3C2A gene expression and clinical variables

All subjects were categorized according to the level of fasting blood glucose^[21]^ as follows: normal glucose group (≤5.6) and hyperglycemia group (>5.6). According to the Chinese age grading standards, all the included subjects were divided into advanced age group (>65) and younger age group (≤65). The results showed that the levels of PIK3C2A mRNA were significantly different between the elderly and the younger groups (*P* = .008). The level of PIK3C2A mRNA was not related to the fasting blood glucose and using anticoagulants. The results are shown in Table 2.

**Table 2:**
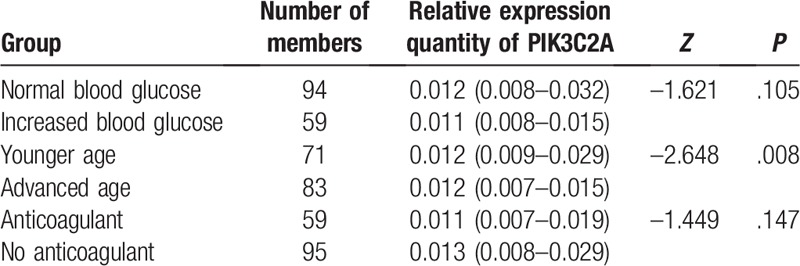
Correlation between expression quantity of PIK3C2A gene and age and fasting blood glucose.

To further examine the correlation between PIK3C2A expression and age, we divided all the studied subjects into the group with high expression quantity (2^−ΔCt^ > 0.0092) and the group with low expression quantity (2^−ΔCt^ ≤ 0.0092) followed by logistic regression analysis. Binary logistic regression analysis indicated that low expression of PIK3C2A was closely related with AMI and the OR value was 2.231. Fasting blood glucose was also closely related with AMI and the OR value was 3.674. However, advanced age was not closely related with AMI (*P* > .05). There was a correlation between low expression and advanced age (rs = −0.451, *P* = .000). See Table 3 for details.

**Table 3:**
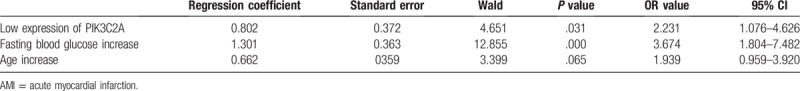
Result of logistic regression analysis on independent risk factors of AMI.

### 3.5 Correlation between relative expression quantity of PIK3C2A gene and severity of coronary artery lesion, cardiac troponin I (TnI)

Gensini score of all the subjects was 40 (15–84.25). The expression quantity of PIK3C2A at the mRNA level was not correlated to Gensini score (rs = −0.013, *P* = .918). Gensini score of cardiac troponin for the AMI group is 0.47 (0.09–7.63) ng/ml. Concentration of serum troponin I (TnI) could reflect the scope of AMI. The expression quantity of PIK3C2A gene in peripheral blood was unrelated to serum TnI concentration (rs = −0.142, *P* = .279).

## 4 Discussion

Coronary heart disease (CHD) was a multifactorial disorder in which inflammation was recognized at all stages of implicated atherosclerosis.^[22]^ Early fatty streaks developed, when the endothelium was activated and expressed chemokines and adhesion molecules, leading to monocyte/lymphocyte recruitment and infiltration into the subendothelium. With the onset of adverse clinical vascular events, the activated cells within the plaque secreted matrix proteases that degraded extracellular matrix proteins and weakened the fibrous cap, resulting in rupture and thrombus formation.^[23]^ In our previous pilot study on profiling gene expression in peripheral blood of AMI patients, we found that AMI patients had a lower expression of PIK3C2A than non-CHD patients. PIK3C2A belongs to phosphoinositide 3-kinases (PI3Ks)—a family of enzymes that phosphorylate the 3′-OH position of the inositol ring of phosphatidylinositol (PI), and regulate a broad range of signaling pathways.^[13]^ It was known that PIK3C2A was same as PI3Kp110b, playing an important role in the regulation of platelet function and thrombosis in arterial blood.^[13,15,24]^ PIK3C2A is structurally distinct from the other members of this class and is expressed in endothelial cells, vascular endothelium, and smooth muscle. In ischemic cardiovascular diseases, such as CAD, pathology is associated with endothelial damage and inflammation, downregulation of the EPC cell population and function, and impaired angiogenesis.^[16]^ It implies that PIK3C2A may play a role in the development and progression of cardiovascular diseases (CADs) by affecting platelet function and impaired angiogenesis.

To test and validate the above hypothesis, we measured and compared levels of PIK3C2A mRNA level in peripheral blood in both AMI and control groups followed by correlation analysis among PIK3C2A, age, fasting blood glucose, and AMI. Our results confirmed that PIK3C2A genes were differentially expressed between the AMI and the control groups. The significant differences of the fasting blood glucose level and patient's age were also found between two groups. However, the abnormal expression of PIK3C2A gene was found to be related to patients’ age but not to fasting blood glucose. The WBC in blood of AMI appears higher than that of the control group. It may be because of the stress of AMI. A experiment using inhibitors of MAPK3/1, PIK3C2A, and RPS6KB1 indicate that all these pathways are important in modulation of proliferation, both at the basal level as well as in response to insulin and oxidative stress.^[25]^ Insulin and oxidative stress share common signal transduction pathways including MAPK3/1 and RPS6KB1. Activation of RPS6KB1 is usually attributed to upstream signals mediated by PIK3C2A.^[25]^ Diabetic DE-gene in the D-EPC-GRN is PIK3C2A, which encodes for the PIK3C2A enzyme that is activated by insulin. Thus, in diabetes PIK3C2A enzyme activity is expected to be suppressed.^[26]^ Glucose uptake is stimulated primarily via PIK3C2A rather than MAPK3/1 or RPS6KB1.^[25]^ Notably, we observed that the abnormal expression of PIK3C2A gene was not found to be related to fasting blood glucose. One reasonable explanation may be that PIK3C2A might promote development of AMI through oxidative stress rather than affecting blood glucose level. Additionally, a recent study showed decreased expression of EPCPik3c2a in coronary artery disease reducing their angiogenic and vasculogenic abilities.^[16]^

Prevalence and fatality rate of AMI was reported to increase with age. Currently, age remained a strong independent predictor of both in-hospital and 1-year post-discharge mortality rates in patients with AMI. But age was not an independent risk factor for AMI (OR 1.939; *P* > .05) in our study. Although it is currently not known whether the PIK3C2A gene contributes to the pathogenesis of AMI, our binary logistic analysis showed that low expression of PIK3C2A gene was an independent risk factor for AMI (OR 2.231; *P* < .05). Regardless of fasting blood glucose and other factors, low expression of PIK3C2A gene alone could increase the risk of AMI by 2.231 times. Also fasting blood glucose increase was an independent risk factor for AMI (OR 3.674; *P* < 0.05). It is low CPNE3 expression that influences AMI—not as much as fasting blood glucose—but it may be a new independent risk factor for AMI.

Gensini scoring has been used to measure coronary artery lesion by using quantitative coronary angiography, in which different weights were applied for varied degrees of severity and the generated scores were closely correlated to degrees of coronary artery stenosis and blood supply.^[27]^ The results of this study demonstrated that the relative expression level of the PIK3C2A gene is uncorrelated to the Gensini score, which indicates the severity of coronary artery lesions. We believe that the expression of PIK3C2A gene cannot be used to predict the degree of coronary artery stenosis. Meanwhile, the expression of PIK3C2A gene is irrelevant to the level of cardiac troponin I while the level of cardiac troponin can reflect the scope of myocardial infarction. We speculate that PIK3C2A may not predict the size of myocardial infarction. Infarct size has been found to be one of the most important determinants of ventricular remodeling.^[28]^ Therefore, the expression of PIK3C2A gene can only be used to predict the risk of AMI, but cannot be used to predict the degree of coronary artery stenosis, the size of myocardial infarction, and even the prognosis. And some studies have shown that, changes in mechanical properties of myocardium caused by a infarction can lead to kinematic abnormalities.^[29]^ A new joint motion feature learning architecture can efficiently establish direct correspondences between motion features and tissue properties and thus can directly acquire the position, shape, and size of an infarction area from a raw CMR sequence.^[29]^ 2D global longitudinal strain, 3D global longitudinal strain, 3D global area strain, and 3D global radial strain were predictive of future LV remodeling after AMI, and 3D global longitudinal strain was an excellent predictor with a highest predictive value among them.^[30]^ In future, the study of PIK3C2A gene may be combined with these findings, and the role of PIK3C2A may be extended from predicting the occurrence of AMI to predicting the size of AMI and even prognosis of patients with AMI.

As known that all atherosclerosis, spasm of coronary artery, thrombosis, immune response, inflammation, especially thrombosis, play the roles in development of AMI, we speculated that PIK3C2A gene did not participate in the process of atherosclerosis and that low expression of PIK3C2A gene might not be caused by AMI neither since PIK3C2A gene was irrelevant to TnI. Instead, PIK3C2A gene might promote the occurrence of acute myocardial infarction by increasing the risk of thrombosis.

As a result, we can reasonably infer that low expression of PIK3C2A gene may promote the occurrence of acute myocardial infarction through oxidative stress. Studies have demonstrated that the excessive activation of lipid peroxidation has a key role in the development of many diseases such as angina and AMI. This is because the lipid peroxidation is a chain of reactions providing a continuous supply of free radicals that increase further peroxidation, A number of studies indicated a link between oxidative stress and AMI.^[31]^ Although it is not sure whether low expression of PIK3C2A causes AMI, we believe that low PIK3C2A expression is one of the causes of AMI.

There are some limitations in our study. First of all, this study is a retrospective study. We observed that low expression of PIK3C2A gene may be a genetic marker for predicting AMI. It would be better to have a prospective study. Secondly, we did not conduct an in-depth study on the characteristics and rules of PIK3C2A gene expression changes at different times.

## 5 Conclusion

The level of PIK3C2A gene expression in patients with AMI is significantly lower than that of healthy people. Low expression of PIK3C2A gene is an independent risk factor of AMI. Low expression of PIK3C2A could serve as a potential biomarker to predict risk of AMI.

## Acknowledgment

The authors thank Professor Zhao Zhihui, School of Animal Husbandry & Veterinary Medicine, Jilin University and his team for their technical guidance in this research.

## Author contributions

**Conceptualization:** Buchuan Tan, Fanbo Meng.

**Data curation:** Buchuan Tan, Miao Liu, Yushuang Yang.

**Formal analysis:** Buchuan Tan, Miao Liu, Long Liu, Fanbo Meng.

**Funding acquisition:** Fanbo Meng.

**Investigation:** Buchuan Tan, Yushuang Yang, Long Liu.

**Methodology:** Yushuang Yang, Fanbo Meng.

**Project administration:** Fanbo Meng.

**Resources:** Miao Liu, Fanbo Meng.

**Software:** Fanbo Meng.

**Writing – Original Draft:** Buchuan Tan.

**Writing – Review & Editing:** Buchuan Tan, Fanbo Meng.
